# A time-calibrated species-level phylogeny of bats (Chiroptera, Mammalia)

**DOI:** 10.1371/currents.RRN1212

**Published:** 2011-02-04

**Authors:** Ingi Agnarsson, Carlos M. Zambrana-Torrelio, Nadia Paola Flores-Saldana, Laura J. May-Collado

**Affiliations:** ^*^University of Puerto Rico, Puerto Rico; ^†^EcoHealth Alliance and ^‡^Asociación para la Biología de la Conservación - Bolivia

## Abstract

Despite their obvious utility, detailed species-level phylogenies are lacking for many groups, including several major mammalian lineages such as bats. Here we provide a cytochrome b genealogy of over 50% of bat species (648 terminal taxa). Based on prior analyzes of related mammal groups, cytb emerges as a particularly reliable phylogenetic marker, and given that our results are broadly congruent with prior knowledge, the phylogeny should be a useful tool for comparative analyzes. Nevertheless, we stress that a single-gene analysis of such a large and old group cannot be interpreted as more than a crude estimate of the bat species tree. Analysis of the full dataset supports the traditional division of bats into macro- and microchiroptera, but not the recently proposed division into Yinpterochiroptera and Yangochiroptera. However, our results only weakly reject the former and strongly support the latter group, and furthermore, a time calibrated analysis of a pruned dataset where most included taxa have the entire 1140bp cytb sequence finds monophyletic Yinpterochiroptera. Most bat families and many higher level groups are supported, however, relationships among families are in general weakly supported, as are many of the deeper nodes of the tree. The exceptions are in most cases apparently due to the misplacement of species with little available data, while in a few cases the results suggest putative problems with current classification, such as the non-monophyly of Mormoopidae. We provide this phylogenetic hypothesis, and an analysis of divergence times, as tools for evolutionary and ecological studies that will be useful until more inclusive studies using multiple loci become available.

## **Introduction**

Phylogenies form the backbone of evolutionary biology and represent tools that underlie a broad spectrum of evolutionary and ecological studies [1] [2]. Phylogenetic work on any given group often first focuses on the ‘big picture’, that is the placement of, and relationship among, major groups, long before species level phylogenies become available. One simple reason for this focus is that general interest questions, such as where and how the major divisions of life fit together, can be answered through sampling relatively few taxa, in a relatively cost and time effective manner. Yet, more detailed species-level phylogenies, often lagging far behind, are the most useful tools for evolutionary and ecological analyses. The above is certainly true for mammalian phylogenetics, where higher level phylogenetics are intensely studied, with the few detailed species level studies for major groups lagging far behind (see e.g. [3] [4] [5] [6]). 

The ultimate goal of phylogenetics must be detailed species level phylogenies of all of life, based on many data. However, achieving this goal will take much time and effort. In the meantime, species level phylogenies may be rapidly reconstructed with already available data using several approaches. One is the construction of phylogenetic supertrees where available trees and taxonomies are united into a summary cladogram [7]. Another is the creation of supermatrices based on available character data. Both approaches make available useful research tools, which may have different strengths. 

The bats (Chiroptera) are one such group where many phylogenetic studies have focused either on understanding higher-level bat relationships (e.g. [8] [9]) or species-level relationships within specific groups (e.g. [10] [11] [12]). Available phylogenies have then been summarized in a supertree [13]. Here, we provide cytochrome b gene tree for over 50% of bat species (648 total taxa). Cytb not only is the most widely available marker for most mammals, but also has been shown to be a particularly reliable phylogenetic marker (e.g. [14]). Thus according with prior analyses of other mammal groups [3] [4] [5] [6], the cytb gene tree can be expected to at least roughly approximate the species-level phylogeny of Chiroptera.  We provide this phylogeny simply as an alternative tool to super-tree phylogenies, until more detailed studies become available. 

##  **Methods**

            Cytochrome b sequences were downloaded from GenBank for 648 bats, including nearly 550 named species, and the remaining terminal taxa being subspecies or unidentified/undescribed species. As outgroups we selected 10 species representing other Pegasoferae [15]: Cetartidoactyla, Perissodactyla, Carnivora, Pholidota (pangolins), and Erinaceomorpha as the primary outgroup. Because many of the taxa have incomplete Cytb sequences, and missing data can cause problems in phylogenetic reconstruction (e.g. [16]), we also created a ‘pruned’ dataset where taxa with less than 30% of the full sequence were removed (‘pruned’ matrix), and another set where only 2 representatives of each family were retained (‘time’ matrix). The latter was used for analysis of divergence times. The sequences were aligned in Mesquite [17], a trivial task given that it is a protein-coding gene with no implied gaps. The appropriate model for the Bayesian analysis was selected with jModeltest [18] using the AIC criterion [19]. The best model was GTR+Γ+I [20] [21]. Bayesian analysis was performed using MrBayes V3.1.2 [22] with settings as in [3] [4] with separate model estimation for first, second, and third codon positions. The MCMC was run with one cold and three heated chains for 30,000,000 generations, sampling trees every 1,000 generations. The first 15,000,000 were then discarded as burnin, after which stationarity was reached. The data matrix and trees are available from the first author and data and trees will be submitted to Treebase (http://www.treebase.org). Genbank accession numbers are listed in Table 1 (see Appendices). 

            The ‘time’ matrix was used to estimate divergence times using relaxed clock methods in BEAST 1.6.1. [23] [24]. For Emballonuridae we additionally retained two *Taphozous* species as these did not group with the other Emballonuridae in the full analysis. The analysis was calibrated using normally distributed priors reflecting: (1) the minimal age of 37 my for the split between Rhinolophidae and Hipposiderids based on the estimated age of the oldest rhinolophid and hipposiderid fossils [25] [26]; (2) the estimated age of Carnivora (split of cat plus dog) of 54 my (the age of Carnivora as estimated by [27]); the estimated age of Chiroptera as a normally distributed prior with mean of 54 my, also based on [27]; and (4) the minimal age of Emballonuridae of 48 my based on the oldest fossils that are with some certainty placed within that family [28]. Prior to the divergence time analysis *Erinaceus* (Erinaceomorpha) and *Talpa* (Eulipotyphla) were set as primary outgroups by enforcing the monophyly of the remaining taxa, and the monophyly of Rhinolopidae plus Hipposideridae was furthermore enforced. The resulting age estimates were then compared to the above mentioned fossil data in addition to the age of other known fossil bats [28]. 

## **Results and Discussion**

### Phylogenetics

The analysis of the full dataset supports the monophyly of bats, and the major division of Chiroptera into Megachiroptera (Pteropodidae) and Microchiroptera with Yangochiroptera contained within the latter group (Figures 1-2).  

**Figure:**
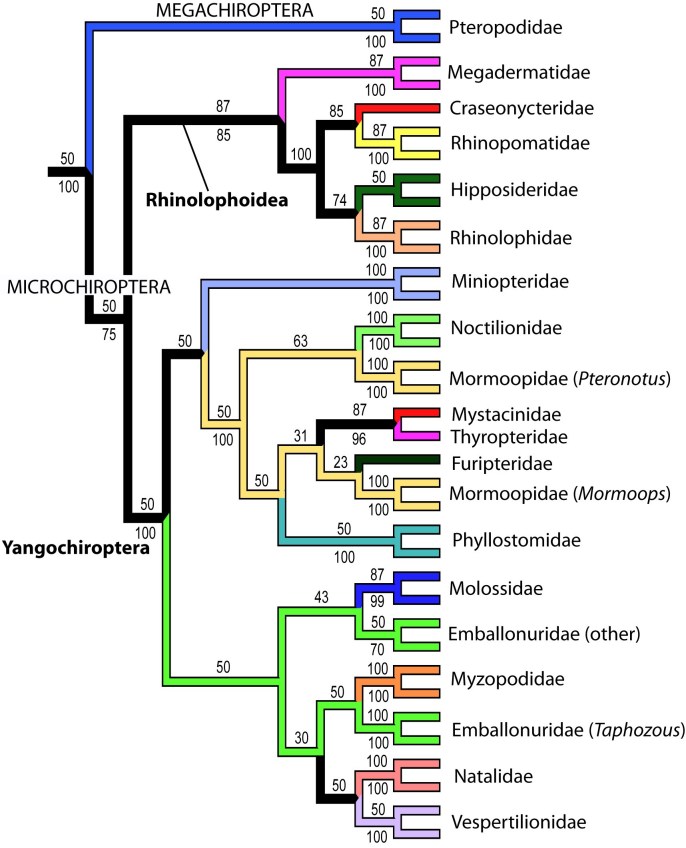
Figure 1. Relationships among bat families according with the analysis of all data. Numbers are posterior probabilities, above branches from the full analysis, below branches support from the pruned analysis.

**Figure:**
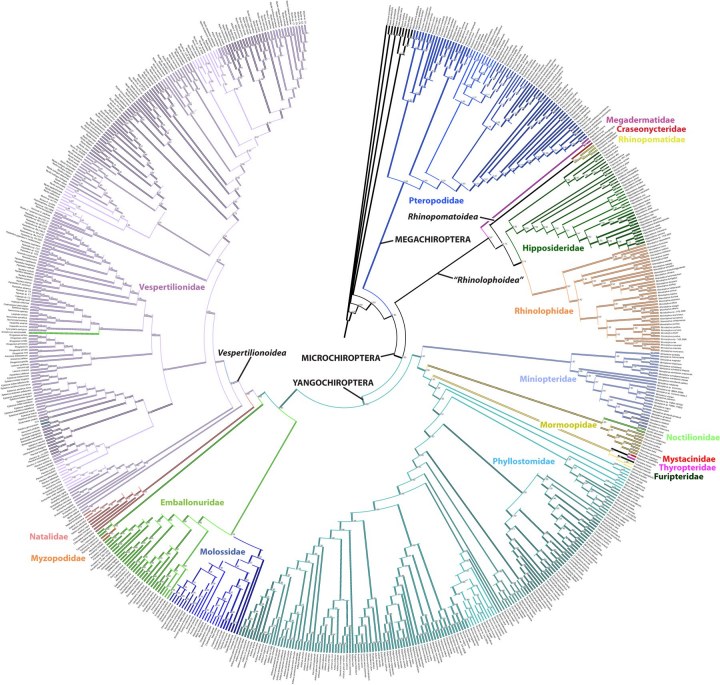
Figure 2. Relationship among bat species with major clade names. Numbers are posterior probabilities. The results are detailed in Figure 4, see Appendices.

The analysis of the ‘time’ matrix, however, supports the now rather generally accepted split into Yinpterochiroptera and Yangochiroptera (see below) (e.g. [29] [30] [31] [32] [33] [34]).

     The Macrochiroptera, or fruitbats (Pteropodidae), are in the main analysis sister to the remaining bats (Figures 2, 4a). Within Pteropodidae most genera are monophyletic, with the exception of *Rousettus angolensis* (synonym *Lissonycteris* *angolensis*) nests with *Myonycteris*. Overall, these results are similar to results of previous studies on macrochiroptera phylogenetics (e.g. [10]).   

The Microchiroptera is divided in two major clades, one is the Yangochiroptera including the families Emballonuridae, Furipteridae, Miniopteridae, Molossidae, Mormoopidae, Mystacinidae, Myzopodidae, Natalidae Noctilionidae, Phyllostomidae, Thyropteridae, and Vespertilionidae. The other major group, which we refer to as a modified “Rhinolophoidea” (Figures 1-2, 4), contains the remaining microbat families Craseonycteridae, Hipposideridae, Megadermatidae, Rhinolophidae, and Rhinopomatidae. Hipposideridae and Rhinolophidae are sister families as supported by previous studies (e.g., [13] [31] [34]).  Only Hipposideridae here contains more than a single genus, and within that family *Hipposiderus* is paraphyletic, containing several small genera. 

 Overall most microchiropteran superfamilies are not supported as monophyletic, except Rhinopomatoidea (Figure 2). A modified Rhinolophoidea that contains Rhinopomatoidea is also supported, and the superfamily Vespertilionioidea is monophyletic except for containing a couple of apparently misplaced species (Figures 2, 4b). The relationships among the families, however, in general are poorly supported and differ among analyses (see Figures 1, 3-4). Taxonomic families are generally recovered either as strictly monophyletic, or approximately, as paraphyletic groups due to one or a couple of ‘misplaced’ taxa.  In the full analysis, families that are strictly supported (i.e. monophyletic, or in the case of families represented by single species, not nesting within another family) are: Craseonycteridae, Furipteridae, Hipposideridae, Megadermatidae, Miniopteridae, Molossidae, Mystacinidae, Myzopodidae, Natalidae, Noctilionidae, Rhinolophidae, Rhinopomatidae, and Thyropteridae. Not monophyletic families are  Phyllostomidae due to the placement of one *Platalina* species nesting within Vestpertilionidae,  Emballonuridae is rendered polyphyletic by the placement of the genus *Taphozous* (2 species) and one species of *Emballonura* outside it. Vespertilionidae is paraphyletic in that within it are placed the above mentioned *Platalina* and *Emballonura*. Finally Mormoopidae forms two clades that are not sister, one including the genus *Mormoops*, the other the genus *Pteronotus*.  These ‘minor’ deviations from family monophyly in most cases probably do not represent refutation of family clades; rather this seems to be mostly an issue of missing data. For example, when species with less than 30% of the sequence are removed, all families are recovered monophyletic, with two exceptions that may be taxonomically informative :(1) The genus *Taphozous* still groups outside Emballonuridae which contradicts previous studies (e.g., [32] [34] [35]) and (2) the Mormoopidae family still forms two separate clades, which agrees with Kennedy et al [36] (for contrasting topologies see e.g., [13] [31]).

Finally, several genera of the family Phyllostomidae are not monophyletic, including *Mimon*, *Mycronycteris*, *Rousettus*, *Vampyressa*, and *Artibeus*. Within Molossidae *Tadarida*, *Mops*, *Chaerephon* are not monophyletic. Within Natalidae, *Chilonatalus* is non-monophyletic, and within Vespertilionidae, the large genera *Pipistrellus* and *Myotis* are not monophyletic.

Many taxa in the full analysis only have available a partial Cytb sequence, and notably clade support is low for many of the deeper clades of the phylogeny. Low support is unsurprising given missing data, and the use of only a single locus for both very many taxa and old divergences. Further, any given gene tree can be expected to differ from the species tree due to various processes including incomplete lineage sorting, introgression, and others. Thus, future effort should focus on resolving the species-level phylogeny of bats with a multi-locus approach. Nevertheless, the phylogeny, especially when the taxa with the highest % missing data are removed, is broadly congruent with prior knowledge, and should thus be a useful tool.   

### Divergence times

The analysis of divergence times (Figure 3) generally agrees with prior studies [27] [35] [37], though the estimated ages are rather lower in general than those estimated by Jones et al. [38].  

**Figure:**
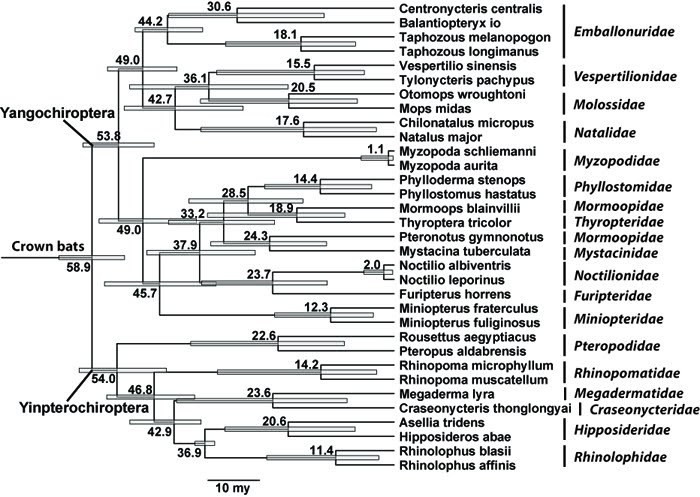
Figure 3. A calibrated phylogeny of bat families. Numbers are in million years, and gray bars are 95% confidence intervals 

In part this may relate to the different suggested relationships among bat families across these studies, though the error margins of many nodes estimated are rather wide and nearly always include age estimates found by prior studies. The results also in most cases are consistent with the available bat fossil record [28]. The age of crown bats, i.e. the split between Yinpterochiroptera and Yangochiroptera is estimated at 58.9 my, a value lying in between the estimates of Cao et al. [27], and Jones et al. [38] and Arnason et al. [37]. Other dates that were included as priors, as expected, also are consistent with the fossil record. The split between Hipposideridae and Rhinolophidae is estimated at 36.9 my, consistent with the oldest known Hipposideridae fossil dated at close to 40 my. Similarly the age of Molossidae estimated at 36.1 my is close to the oldest Molossidae fossil at near 40 my [28]. The split between Emballonuridae and its sister lineage is estimated at 49 my, right around the age of the oldest emballonurid fossil. Most other dates are also consistent with the fossil record. The genus *Taphozous*has a fossil record going up to 20.4 my, a date in between the estimated split between crown *Taphozous* (18.1 my) and the split between *Taphozous* and other Emballonuridae (44.2 my). The oldest Mystacinidae fossil dates from around 20 my [28] and the estimated split here between Mystacinidae and its sister lineage is 24.3 my. The oldest Phyllostomidae fossil dates from around 16 my [28], a date in between the split between crown *Phyllostoma* (14.4 my) and the split between Phyllostomidae and its sister lineage (28.5 my). In a few cases the estimates are younger than possible given current understanding the fossil record, e.g. the age of Megadermatidae at 23.6 my while the oldest fossil is at least 37 my. However, 95% confidence interval of this node estimate reaches over 40 my. The age of Natalidae, estimated at around 43 my, is younger than the oldest fossil thought to belong to that family, at over 50 my. Similarly one putative Vespertilionidae genus, *Stehlinia*, has a fossil record older (up to 48 my) than the estimated age of the family at 36.1 my. These mismatches may reflect simply erroneous age estimates, or could possibly indicate that some fossil bats are taxonomically misplaced. In most other cases the estimated ages are older than the oldest available fossils, which may reflect the incompleteness of the fossil record. 

In sum, we provide a *cytochrome b* genealogy for Chiroptera, which we expect to crudely approximate the bat species tree. Until more detailed species-level phylogenies become available, this offers an alternative phylogenetic tool to super-tree phylogenies, for comparative evolutionary, ecological analyzes, and phylogenetic conservation assessment.

## **Acknowledgments**

Thanks to PLoS Currents: Tree of Life board of reviewers, the editor, and two anonymous reviewers for comments that improved this manuscript.

**Funding information**

This research was funded, in part, by the University of Puerto Rico. ** Competing interests **The authors have declared that no competing interests exist.

**Appendices**

**Figure 4. **Results from Fig. 2 in standard tree format.

**Figure:**
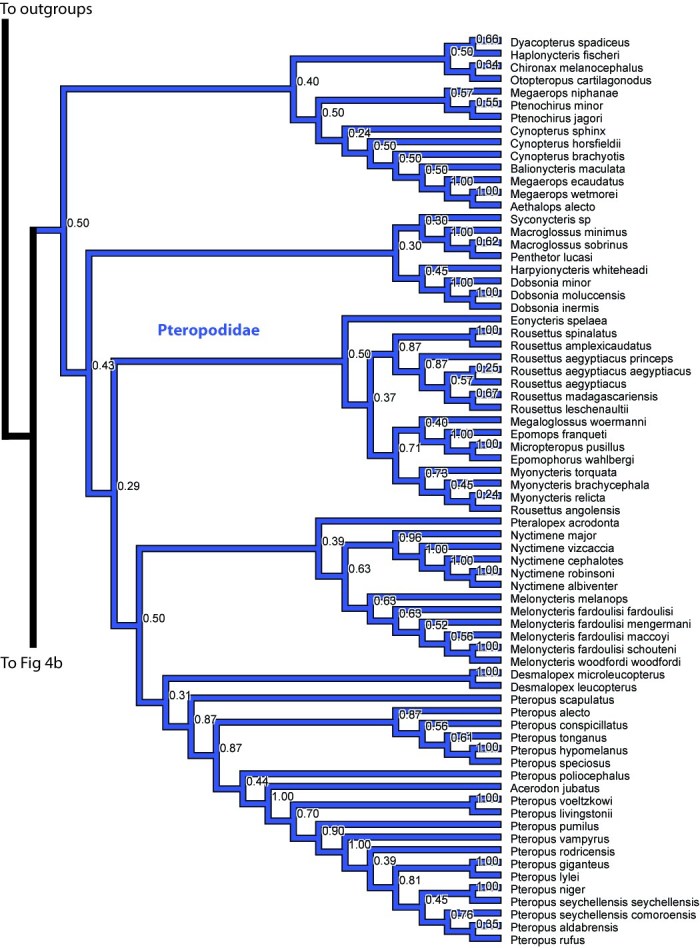
Figure 4a. Results from Figure 2, Pteropodidae. Numbers are posterior probabilities

**Figure:**
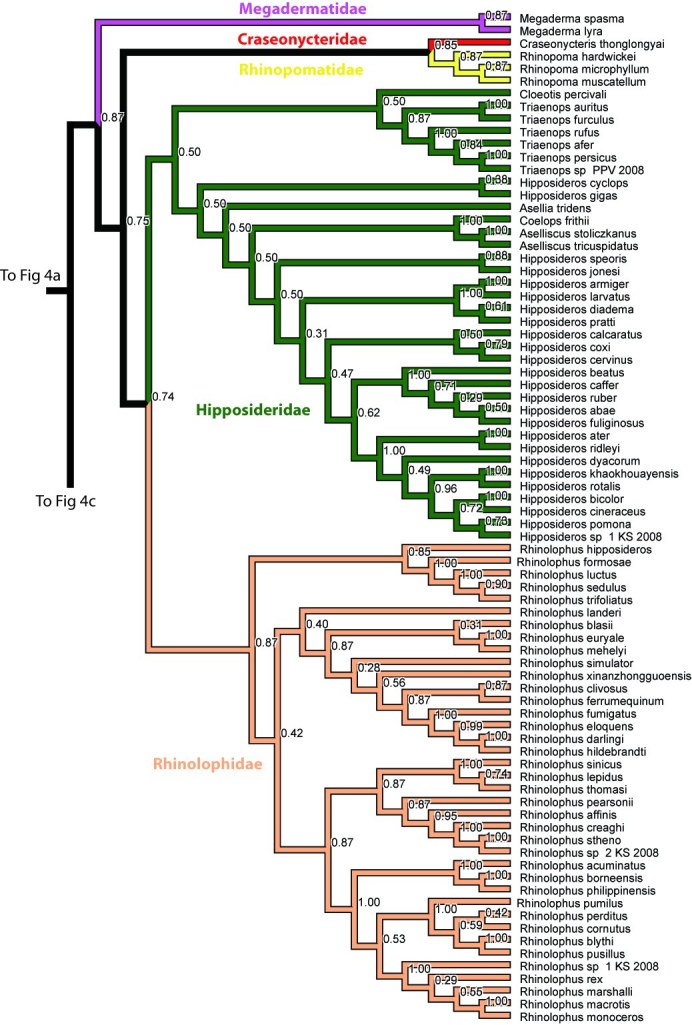
Figure 4b. Results from Figure 2, Megadermatidae, Craseonycteridae, Rhinopomatidae, Hipposideridae, and Rhinolophidae. Numbers are posterior probabilities.

**Figure:**
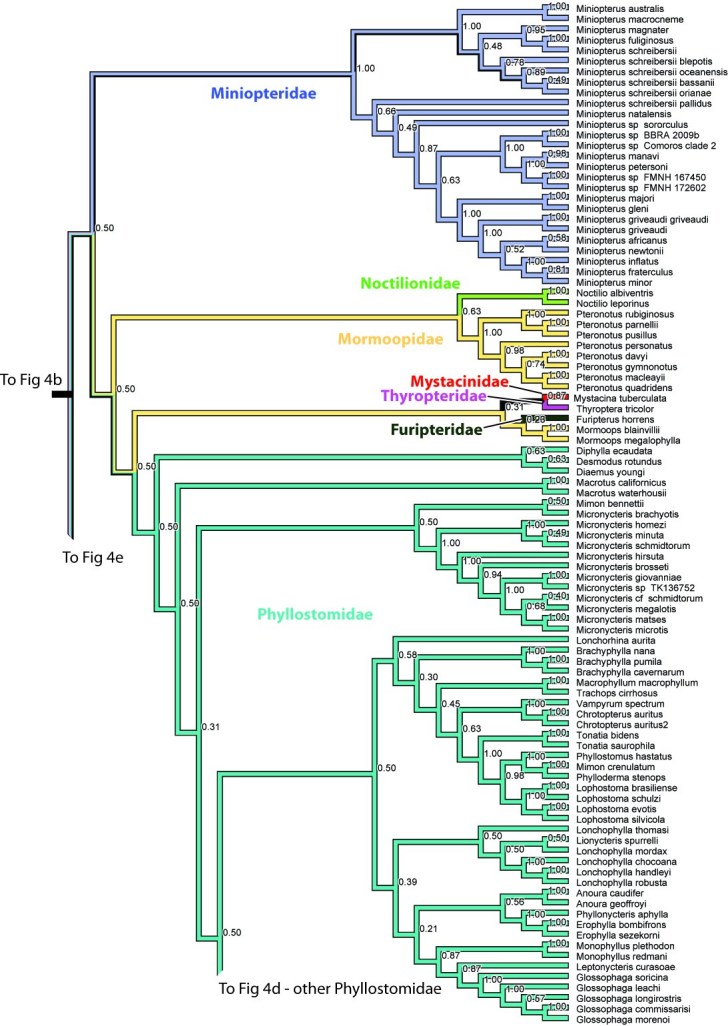
Figure 4c. Results from Figure 2, Miniopteridae, Noctilionidae, Mormoopidae, Mystacinidae, Thyropteridae, Furipteridae, and Phyllostomidae in part. Numbers are posterior probabilities

**Figure:**
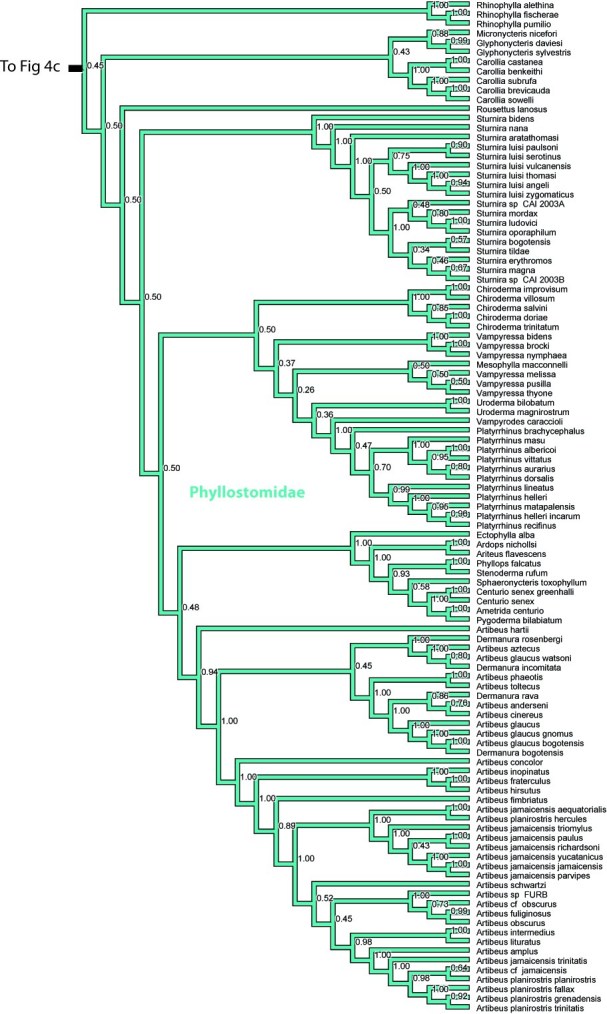
Figure 4d. Results from Figure 2, Phyllostomidae, in part. Numbers are posterior probabilities

**Figure:**
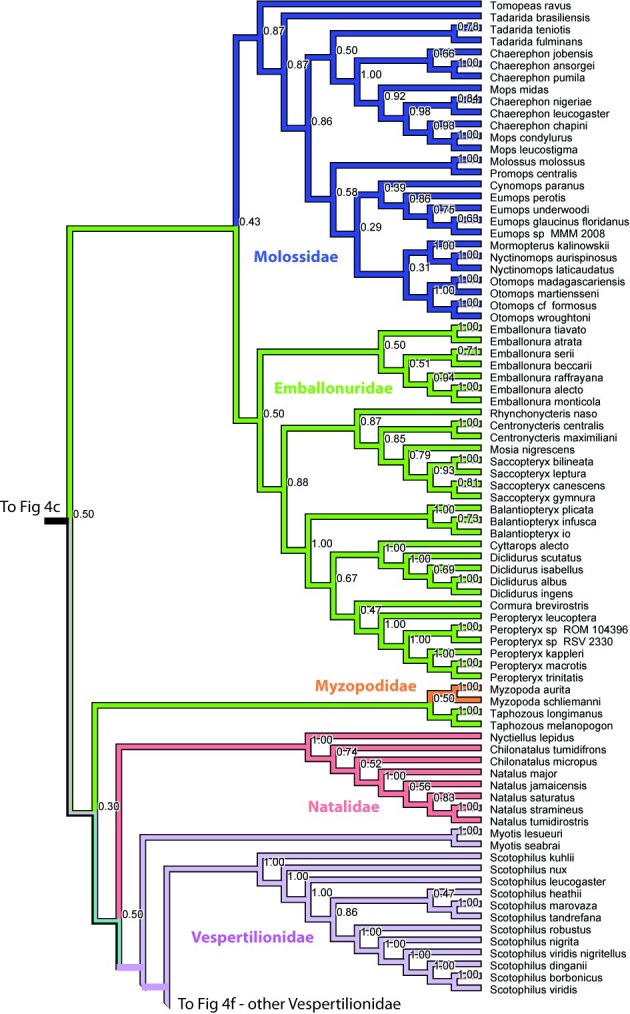
Figure 4e. Results from Figure 2, Molossidae,Emballonuridae, Myzopodidae, Natalidae, and Vespertilionidae in part. Numbers are posterior probabilities

**Figure:**
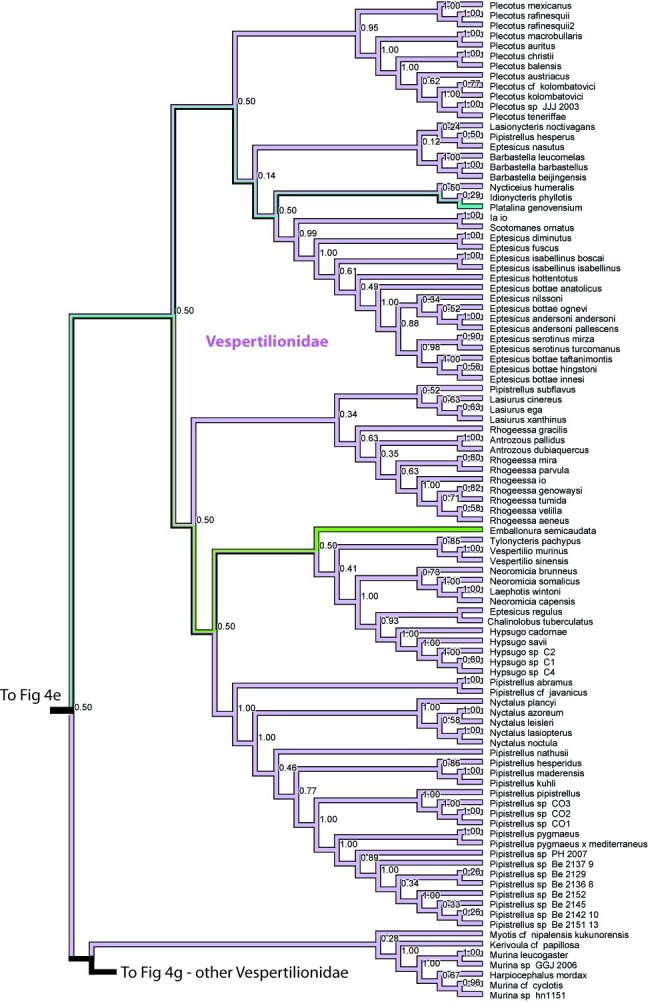
Figure 4f. Results from Figure 2, Vespertilionidae in part. Numbers are posterior probabilities

**Figure:**
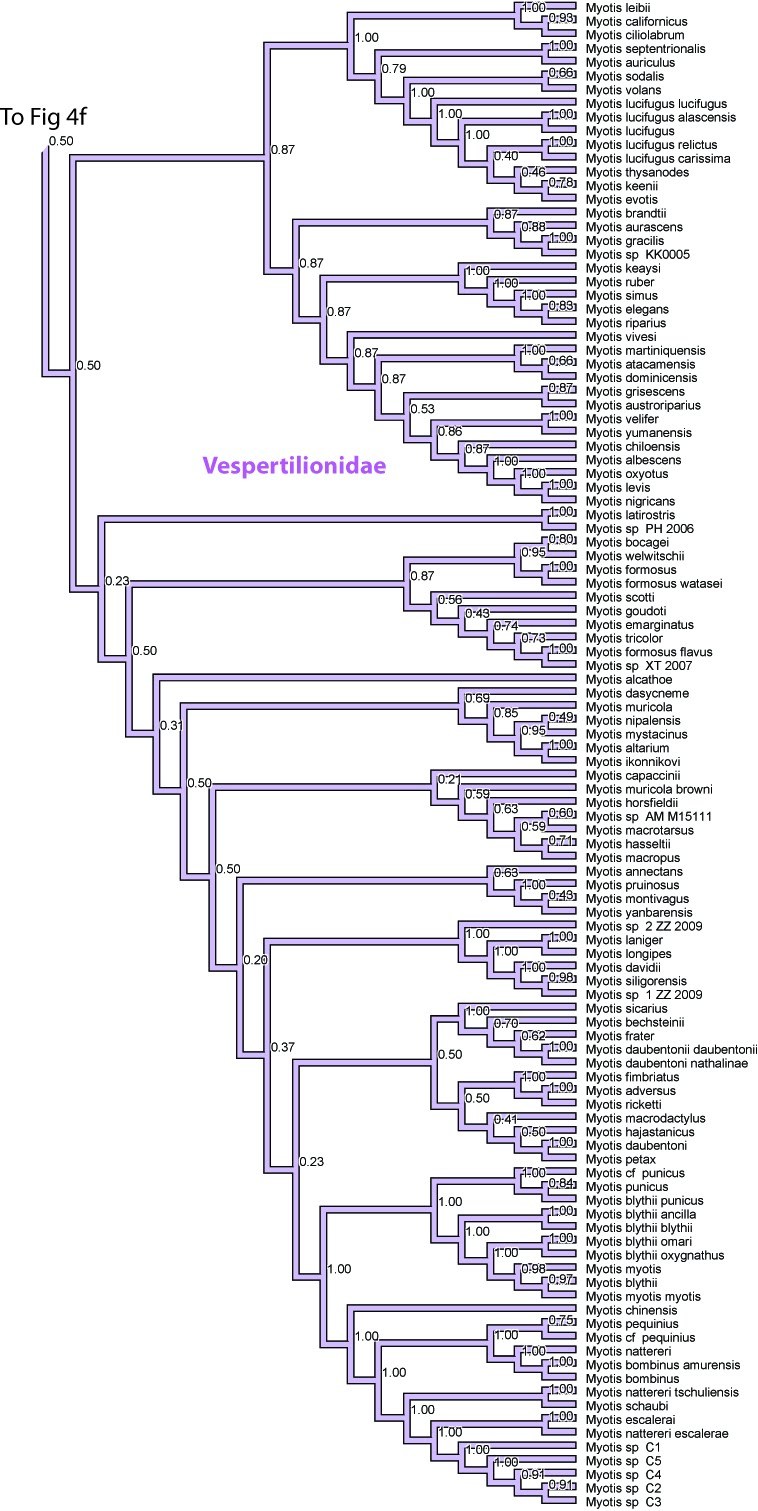
Figure 4g. Results from Figure 2, Vespertilionidae in part. Numbers are posterior probabilities

****

**Table 1.**

Species included and Genbank accession numbers

**Table:** 

Genus	Species	sub sp or voucher	Accession Number	
Acerodon	jubatus		EU330962	
Aethalops	alecto		AY629006	
Ametrida	centurio		AY604446	
Anoura	caudifer		L19506	
Anoura	geoffroyi		FJ155495	
Antrozous	dubiaquercus		EF222381	
Antrozous	pallidus		EF222382	
Ardops	nichollsi		AY572337	
Ariteus	flavescens		AY604436	
Artibeus	amplus		EU160947	
Artibeus	anderseni		U66509	
Artibeus	aztecus		U66510	
Artibeus	cf.jamaicensis		DQ985486	
Artibeus	cf.obscurus		DQ903818	
Artibeus	cinereus		EU805599	
Artibeus	concolor		U66519	
Artibeus	fimbriatus		U66498	
Artibeus	fraterculus		U66499	
Artibeus	fuliginosus		L19505	
Artibeus	glaucus	watsoni	FJ179259	
Artibeus	glaucus		U66512	
Artibeus	glaucus	bogotensis	EU805596	
Artibeus	glaucus	gnomus	EU805594	
Artibeus	hartii		EU160972	
Artibeus	hirsutus		U66500	
Artibeus	inopinatus		U66501	
Artibeus	intermedius		FJ179231	
Artibeus	jamaicensis	aequatorialis	DQ869450	
Artibeus	jamaicensis	jamaicensis	DQ869518	
Artibeus	jamaicensis	parvipes	DQ869474	
Artibeus	jamaicensis	paulus	DQ869456	
Artibeus	jamaicensis	richardsoni	DQ869454	
Artibeus	jamaicensis	trinitatis	DQ003028	
Artibeus	jamaicensis	triomylus	AY382782	
Artibeus	jamaicensis	yucatanicus	DQ869484	
Artibeus	lituratus		EU160813	
Artibeus	obscurus		U66507	
Artibeus	phaeotis		FJ376727	
Artibeus	planirostris	fallax	DQ869426	
Artibeus	planirostris	grenadensis	DQ869439	
Artibeus	planirostris	hercules	DQ869421	
Artibeus	planirostris	planirostris	DQ869396	
Artibeus	planirostris	trinitatis	DQ869433	
Artibeus	schwartzi		DQ869531	
Artibeus	sp.FURB		DQ985497	
Artibeus	toltecus		U66515	
Asellia	tridens		FJ457617	
Aselliscus	stoliczkanus		EU434954	
Aselliscus	tricuspidatus		DQ888679	
Balantiopterys	infusca		EF584151	
Balantiopterys	io		EF584153	
Balantiopterys	plicata		EF584154	
Balionycteris	maculata		AF044636	
Barbastella	barbastellus		EU360700	
Barbastella	beijingensis		EF534762	
Barbastella	leucomelas		EF534766	
Brachyphylla	cavernarum		AY572383	
Brachyphylla	nana		EU521680	
Brachyphylla	pumila		EU521678	
Carollia	benkeithi		DQ177282	
Carollia	brevicauda		FJ154120	
Carollia	castanea		DQ888289	
Carollia	sowelli		AF511973	
Carollia	subrufa		AF187024	
Centronycteris	centralis		EF584155	
Centronycteris	maximiliani		EF584157	
Centurio	senex	greenhalli	AY604445	
Centurio	senex		AY604444	
Chaerephon	ansorgei		AY377967	
Chaerephon	chapini		AY591329	
Chaerephon	jobensis		AY591331	
Chaerephon	leucogaster		EU716041	
Chaerephon	nigeriae		AY591330	
Chaerephon	pumila		AY614756	
Chalinolobus	tuberculatus		NC_002626	
Chilonatalus	micropus		AY621026	
Chilonatalus	tumidifrons		AY621028	
Chiroderma	improvisum		L28938	
Chiroderma	doriae		AY169958	
Chiroderma	salvini		L28939	
Chiroderma	trinitatum		DQ312413	
Chiroderma	villosum		FJ154121	
Chironax	melanocephalus		AY629005	
Chrotopterus	auritus		FJ155481	
Cloeotis	percivali		FJ457616	
Coelops	frithii		EU434955	
Cormura	brevirostris		EF584159	
				
Craseonycteris	thonglongyai		EF035012	
Cynomops	paranus		AY675219	
Cynopterus	brachyotis		EF201644	
Cynopterus	horsfieldii		EF201643	
Cynopterus	sphinx		DQ445703	
Cyttarops	aleco		EF584162	
Dermanura	bogotensis		FJ376714	
Dermanura	rava		FJ179252	
Dermanura	rosenbergi		FJ179254	
Dermanura	incomitata		FJ376718	
Desmalopex	leucopterus		EU330965	
Desmalopex	microleucopterus		EU330976	
Desmodus	rotundus		FJ155477	
Diaemus	youngi		FJ155475	
Diclidurus	albus		EF584163	
Diclidurus	ingens		EF584164	
Diclidurus	isabellus		EF584166	
Diclidurus	scutatus		EF584167	
Diphylla	ecaudata		FJ155476	
Dobsonia	inermis		DQ445704	
Dobsonia	minor		DQ445705	
Dobsonia	moluccensis		AF144064	
Dyacopterus	spadiceus		EF105531	
Ectophylla	alba		DQ312404	
Emballonura	alecto		AY426101	
Emballonura	atrata		DQ178261	
Emballonura	beccarrii		EF584222	
Emballonura	monticola		EF584223	
Emballonura	raffrayana		EF584224	
Emballonura	semicaudata		EF635553	
Emballonura	serii		EF635544	
Emballonura	tiavato		DQ178285	
Eonycteris	spelaea		AB062476	
Epomophorus	wahlbergi		DQ445706	
Epomops	franqueti		DQ445707	
Eptesicus	andersoni	andersoni	EU786850	
Eptesicus	andersoni	pallescens	EU786841	
Eptesicus	bottae	anatolicus	EU786812	
Eptesicus	bottae	hingstoni	EU786819	
Eptesicus	bottae	innesi	EU786815	
Eptesicus	bottae	ogveni	EU786876	
Eptesicus	bottae	taftanimontis	EU786814	
Eptesicus	diminutus		EU786864	
Eptesicus	fuscus		EU786866	
Eptesicus	hottentotus		EU786823	
Eptesicus	isabellinus	boscai	EU786838	
Eptesicus	isabellinus	isabellinus	EU786831	
Eptesicus	nasutus		EU786840	
Eptesicus	nilssoni		AF376836	
Eptesicus	regulus		AY007531	
Eptesicus	serotinus	mirza	EU786861	
Eptesicus	serotinus	turcomanus	EU786875	
Erophylla	bombifrons		AY620438	
Erophylla	sezekorni		AY620439	
Eumops	glaucinus	floridanus	EU350026	
Eumops	perotis		EU349991	
Eumops	sp.	MMM-2008	EU349999	
Eumops	underwoodi		EU349989	
Furipterus	horrens		AY621004	
Glossophaga	commissarisi		AF382886	
Glossophaga	leachi		AF382878	
Glossophaga	longirostris		AF382875	
Glossophaga	morenoi		AF382882	
Glossophaga	soricina		FJ392516	
Glyphonycteris	daviesi		AY380747	
Glyphonycteris	sylvestris		AY380746	
Haplonycteris	fischeri		AY817881	
Harpiocephalus	mordax		AJ841971	
Harpyionycteris	whiteheadi		DQ445708	
Hipposideros	abae		EU934448	
Hipposideros	armiger		EU434946	
Hipposideros	ater		DQ054807	
Hipposideros	beatus		FJ347976	
Hipposideros	bicolor		DQ054808	
Hipposideros	caffer		FJ347980	
Hipposideros	calcaratus		DQ054806	
Hipposideros	cervinus		DQ054805	
Hipposideros	cineraceus		DQ054809	
Hipposideros	coxi		EF108148	
Hipposideros	cyclops		EU934466	
Hipposideros	diadema		DQ219421	
Hipposideros	dyacorum		EF108151	
Hipposideros	fuliginosus		EU934468	
Hipposideros	gigas		EU934470	
Hipposideros	jonesi		EU934473	
Hipposideros	khaokhouayensis		DQ054816	
Hipposideros	larvatus		EU434949	
Hipposideros	pomona		EU434950	
Hipposideros	pratti		EU434952	
Hipposideros	ridleyi		DQ054812	
Hipposideros	rotalis		DQ054814	
Hipposideros	ruber		FJ347996	
Hipposideros	sp.1KS-2008		EU434948	
Hipposideros	speoris		DQ680823	
Hypsugo	cardonae		DQ318883	
Hypsugo	savii		DQ120866	
Hypsugo	sp.C1		EU360677	
Hypsugo	sp.C2		EU360678	
Hypsugo	sp.C4		EU360679	
Ia	io		DQ302094	
Idionycteris	phyllotis		IINMTCYTB	
Kerivoula	cf.papillosa		AJ841970	
Laephotis	wintoni		AJ841964	
Lasionycteris	noctivagans		LSNMTCYTBZ	
Lasiurus	cinereus		DQ421825	
Lasiurus	ega		DQ421826	
Lasiurus	xanthinus		AF369549	
Leptonycteris	curasoae		AF382889	
Lionycteris	spurrelli		AF423100	
Lonchophylla	chocoana		AF423092	
Lonchophylla	handleyi		AF423094	
Lonchophylla	mordax		AF423095	
Lonchophylla	robusta		AF423091	
Lonchophylla	thomasi		AF423086	
Lonchorhina	aurita		FJ155494	
Lophostoma	silvicola		FJ155493	
Lophostoma	brasiliense		FJ155486	
Lophostoma	evotis		FJ155491	
Lophostoma	schulzi		FJ155485	
Macroglossus	minimus		AY926645	
Macroglossus	sobrinus		FJ226494	
Macrophyllum	macrophyllum		FJ155484	
Macrotus	californicus		AY380744	
Macrotus	waterhousii		AY380745	
Megaderma	lyra		DQ888678	
Megaderma	spasma		AY057942	
Megaerops	ecaudatus		EF201645	
Megaerops	niphanae		AF044647	
Megaerops	wetmorei		EF105537	
Megaloglossus	woermanni		DQ445710	
Melonycteris	fardoulisi	fardoulisi	AY847251	
Melonycteris	fardoulisi	maccoyi	AY847254	
Melonycteris	fardoulisi	mengermani	AY847241	
Melonycteris	fardoulisi	schouteni	AY847236	
Melonycteris	melanops		AF044645	
Melonycteris	woodfordi	woodfordi	AY847234	
Mesophylla	macconnelli		FJ154122	
Micronycteris	brachyotis		AY380748	
Micronycteris	brosseti		AY380771	
Micronycteris	cf.schmidtorum		DQ077407	
Micronycteris	giovanniae		AY380750	
Micronycteris	hirsuta		DQ077415	
Micronycteris	homezi		AY380754	
Micronycteris	matses		DQ077419	
Micronycteris	megalotis		DQ077429	
Micronycteris	microtis		AY380756	
Micronycteris	minuta		DQ077405	
Micronycteris	schmidtorum		DQ077442	
Micronycteris	sp.TK136752		DQ077420	
Micronycteris	nicefori		AY380749	
Micropteropus	pusillus		AF044648	
Mimon	crenulatum		FJ155478	
Mimon	bennettii		DQ903832	
Miniopterus	africanus		EF363524	
Miniopterus	australis		AY614735	
Miniopterus	fraterculus		AJ841975	
Miniopterus	fuliginosus		AB085735	
Miniopterus	gleni		FJ383146	
Miniopterus	griveaudi		FJ232802	
Miniopterus	griveaudi	griveaudi	FJ383143	
Miniopterus	inflatus		AY614737	
Miniopterus	macrocneme		AY614734	
Miniopterus	magnater		EF517308	
Miniopterus	majori		DQ899776	
Miniopterus	manavi		FJ383130	
Miniopterus	minor		FJ232805	
Miniopterus	natalensis		AY614744	
Miniopterus	newtonii		EF363521	
Miniopterus	petersoni		EU091258	
Miniopterus	pusillus		DQ837650	
Miniopterus	schreibersii		EF530348	
Miniopterus	schreibersii	bassanii	AY614733	
Miniopterus	schreibersii	blepotis	AF217444	
Miniopterus	schreibersii	oceanensis	AF130123	
Miniopterus	schreibersii	orianae	AY614732	
Miniopterus	schreibersii	pallidus	AY614736	
Miniopterus	sp.	BBRA-2009b	FJ383134	
Miniopterus	sp.	Comoros clade 2	FJ232800	
Miniopterus	sp.	FMNH 167450	FJ383132	
Miniopterus	sp.	FMNH 172602	FJ383133	
Miniopterus	sp.	sororculus	DQ899771	
Molossus	molossus		L19724	
Monophyllus	plethodon		AF382887	
Monophyllus	redmani		AF382888	
Mops	condylurus		EF474030	
Mops	leucostigma		EF474029	
Mops	midas		EF474049	
Mormoops	blainvillii		AY604462	
Mormoops	megalophylla		AF330808	
Mormopterus	kalinowskii		L19725	
Mosia	nigrescens		EF635558	
Murina	cf.cyclotis		AJ841974	
Murina	leucogaster		AB085733	
Murina	sp.	GGJ-2006	DQ435071	
Murina	sp.	hn1151	EF570883	
Myonycteris	brachycephala		AF044644	
Myonycteris	relicta		AF044649	
Myonycteris	torquata		AF044650	
Myotis	adversus		AB106587	
Myotis	albescens		AF376839	
Myotis	alcathoe		AJ841955	
Myotis	altarium		FJ215677	
Myotis	annectans		AJ841956	
Myotis	atacamensis		AM261882	
Myotis	aurascens		AY665161	
Myotis	auriculus		AM261884	
Myotis	austroripari		AM261885	
Myotis	bechsteinii		AF376843	
Myotis	blythii		DQ120906	
Myotis	blythii	ancilla	AM284170	
Myotis	blythii	blythii	AF376840	
Myotis	blythii	omari	DQ288853	
Myotis	blythii	oxygnathus	AF376841	
Myotis	blythii	punicus	AF376842	
Myotis	bocagei		AJ504408	
Myotis	bombinus		EF555240	
Myotis	bombinus	amurensis	AM284169	
Myotis	brandtii		AM261886	
Myotis	californicus		AM261887	
Myotis	capaccinii		AF376845	
Myotis	cf.nipalensis	kukunorensis	AY699845	
Myotis	cf.pequinius		AM284173	
Myotis	cf.punicus		AF246252	
Myotis	chiloensis		AM261888	
Myotis	chinensis		AB106588	
Myotis	ciliolabrum		AM261889	
Myotis	dasycneme		AF376846	
Myotis	daubentoni		AY665137	
Myotis	daubentoni	nathalinae	AF376862	
Myotis	daubentoni	daubentonii	EU153105	
Myotis	davidii		AB106591	
Myotis	dominicensis		AF376848	
Myotis	elegans		AM261891	
Myotis	emarginatus		AF376849	
Myotis	escalerai		FJ460363	
Myotis	evotis		AJ841949	
Myotis	fimbriatus		EF555226	
Myotis	formosus		AJ841950	
Myotis	formosus	flavus	EU434932	
Myotis	formosus	watasei	EU434933	
Myotis	frater		AB106593	
Myotis	goudoti		AJ504451	
Myotis	gracilis		AB243029	
Myotis	grisescens		AM261892	
Myotis	hajastanicus		AY665138	
Myotis	hasseltii		AF376850	
Myotis	horsfieldii		AF376851	
Myotis	ikonnikovi		AB106602	
Myotis	keaysi		AF376852	
Myotis	keenii		AM262329	
Myotis	laniger		EF555229	
Myotis	latirostris		AM262330	
Myotis	leibii		AM262331	
Myotis	lesueuri		AY485687	
Myotis	levis		AF376853	
Myotis	longipes		FJ215678	
Myotis	lucifugus		AF376854	
Myotis	lucifugus	alascensis	DQ503483	
Myotis	lucifugus	carissima	AF294512	
Myotis	lucifugus	lucifugus	DQ503488	
Myotis	lucifugus	relictus	DQ503558	
Myotis	macrodactylus		EF555238	
Myotis	macropus		AJ841959	
Myotis	macrotarsus		AJ841960	
Myotis	martiniquensis		AM262332	
Myotis	montivagus		AM262333	
Myotis	muricola		AY665144	
Myotis	muricola	browni	AF376859	
Myotis	myotis		AM261883	
Myotis	myotis	myotis	AF246246	
Myotis	mystacinus		AY665167	
Myotis	nattereri		AB106606	
Myotis	nattereri	escalerae	EU360649	
Myotis	nattereri	tschuliensis	AM284171	
Myotis	nigricans		AF376864	
Myotis	nipalensis		AY699844	
Myotis	oxyotus		AF376865	
Myotis	pequinius		AM284172	
Myotis	petax		EF555236	
Myotis	pruinosus		AB106607	
Myotis	punicus		EU360640	
Myotis	ricketti		AJ504452	
Myotis	riparius		AF376866	
Myotis	ruber		AF376867	
Myotis	schaubi		AF376868	
Myotis	scotti		AJ841958	
Myotis	seabrai		AJ841962	
Myotis	septentrionalis		AM262335	
Myotis	sicarius		AJ841951	
Myotis	siligorensis		FJ215679	
Myotis	simus		AM262336	
Myotis	sodalis		AM262337	
Myotis	sp.	1 ZZ-2009	FJ215680	
Myotis	sp.	2 ZZ-2009	FJ215681	
Myotis	sp.	AM_M15111	AY007527	
Myotis	sp.	C1	EU360644	
Myotis	sp.	C2	EU360645	
Myotis	sp.	C3	EU360646	
Myotis	sp.	C4	EU360647	
Myotis	sp.	C5	EU360648	
Myotis	sp.	KK0005	AB106609	
Myotis	sp.	PH-2006	DQ337479	
Myotis	sp.	XT-2007	EF555233	
Myotis	thysanodes		AF376869	
Myotis	tricolor		AJ841952	
Myotis	velifer		AF376870	
Myotis	vivesi		AJ504406	
Myotis	volans		AF376871	
Myotis	welwitschii		AF376874	
Myotis	yanbarensis		AB106610	
Myotis	yumanensis		AF376875	
Mystacina	tuberculata		AY960981	
Myzopoda	aurita		EF432190	
Myzopoda	schliemanni		EF432213	
Natalus	jamaicensis		AY621023	
Natalus	major		AY621021	
Natalus	saturatus		AY621014	
Natalus	stramineus		AY621019	
Natalus	tumidirostris		AY621008	
Neoromicia	brunneus		EU786868	
Neoromicia	capensis		AJ841966	
Neoromicia	somalicus		EU786869	
Noctilio	albiventris		AF330806	
Nyctalus	azoreum		DQ887590	
Nyctalus	lasiopterus		DQ120871	
Nyctalus	leisleri		AF376832	
Nyctalus	noctula		AJ841967	
Nyctalus	plancyi		DQ435073	
Nycteris	leporinus		AF330802	
Nycticeius	humeralis		L19727	
Nyctiellus	lepidus		AY621007	
Nyctimene	albiventer		DQ314264	
Nyctimene	cephalotes		DQ314268	
Nyctimene	major		AF044652	
Nyctimene	robinsoni		AF144066	
Nyctimene	vizcaccia		DQ445711	
Nyctinomops	aurispinosus		L19728	
Nyctinomops	laticaudatus		L19729	
Otomops	cf.formosus		EF504252	
Otomops	madagascariensis		EF216381	
Otomops	martiensseni		EF216441	
Otomops	wroughtoni		EF504251	
Otopteropus	cartilagonodus		AY974770	
Penthetor	lucasi		EF105542	
Peropteryx	kappleri		EF584169	
Peropteryx	leucoptera		EF584175	
Peropteryx	macrotis		EF584180	
Peropteryx	spvoucherROM104396		EF584170	
Peropteryx	spvoucherRSV2330		EF584171	
Peropteryx	trinitatis		EF584182	
Phylloderma	stenops		FJ155480	
Phyllonycteris	aphylla		AF187033	
Phyllops	falcatus		DQ211651	
Phyllostomus	hastatus		FJ155479	
Pipistrellus	abramus		AJ504448	
Pipistrellus	cf.javanicus		AJ504447	
Pipistrellus	hesperidus		AJ841968	
Pipistrellus	hesperus		DQ421823	
Pipistrellus	kuhli		AJ504444	
Pipistrellus	maderensis		AJ426632	
Pipistrellus	nathusii		AJ504446	
Pipistrellus	pipistrellus		AJ504443	
Pipistrellus	pygmaeus		DQ120856	
Pipistrellus	pygmaeusxmediterraneus		AJ504442	
Pipistrellus	sp.	Be_2136_8	AY426091	
Pipistrellus	sp.	Be_2137_9	AY426092	
Pipistrellus	sp.	Be_2142_10	AY426089	
Pipistrellus	sp.	Be_2145	AY316334	
Pipistrellus	sp.	Be_2151_13	AY426090	
Pipistrellus	sp.	Be_2152	AY316332	
Pipistrellus	sp.	CO1	EU420890	
Pipistrellus	sp.	CO2	EU420891	
Pipistrellus	sp.	CO3	EU420892	
Pipistrellus	sp.	PH-2007	EF370417	
Pipistrellus	sp.	Be_2129	AY316333	
Pipistrellus	subflavus		AJ504449	
Platalina	genovensium		AF423101	
Platyrrhinus	albericoi		FJ154124	
Platyrrhinus	helleri		FJ154141	
Platyrrhinus	helleri	incarum	FJ154146	
Platyrrhinus	masu		FJ154164	
Platyrrhinus	matapalensis		FJ154168	
Platyrrhinus	aurarius		FJ154127	
Platyrrhinus	brachycephalus		FJ154132	
Platyrrhinus	dorsalis		FJ154139	
Platyrrhinus	lineatus		FJ154160	
Platyrrhinus	recifinus		FJ154176	
Platyrrhinus	vittatus		FJ154178	
Plecotus	auritus		EF570882	
Plecotus	austriacus		EU360707	
Plecotus	balensis		AF513798	
Plecotus	cf.kolombatovici		AF513783	
Plecotus	christii		EU743801	
Plecotus	kolombatovici		AF513785	
Plecotus	macrobullaris		AF513805	
Plecotus	mexicanus		AY776038	
Plecotus	rafinesquii		AY776084	
Plecotus	sp.	JJJ-2003	AF513791	
Plecotus	teneriffae		EU360704	
Promops	centralis		L19732	
Ptenochirus	jagori		AB046325	
Ptenochirus	minor		AY974702	
Pteralopex	acrodonta		FJ561376	
Pteronotus	davyi		AF338672	
Pteronotus	gymnonotus		AF338675	
Pteronotus	macleayii		AY604461	
Pteronotus	parnellii		AY604456	
Pteronotus	personatus		AF338680	
Pteronotus	pusillus		AY604455	
Pteronotus	quadridens		AY604460	
Pteronotus	rubiginosus		AY604457	
Pteropus	rufus		AB085732	
Pteropus	aldabrensis		FJ561394	
Pteropus	alecto		AF144065	
Pteropus	conspicillatus		FJ561380	
Pteropus	giganteus		FJ561381	
Pteropus	hypomelanus		FJ561383	
Pteropus	livingstonii		FJ561384	
Pteropus	lylei		EF584229	
Pteropus	niger		FJ561385	
Pteropus	poliocephalus		FJ561387	
Pteropus	pumilus		FJ561390	
Pteropus	rodricensis		FJ561392	
Pteropus	scapulatus		FJ561377	
Pteropus	seychellensis	seychellensis	FJ561399	
Pteropus	seychellensis	comoroensis	FJ561398	
Pteropus	speciosus		AB062474	
Pteropus	tonganus		AF044656	
Pteropus	vampyrus		FJ561401	
Pteropus	voeltzkowi		FJ561405	
Pygoderma	bilabiatum		AY604438	
Rhinolophus	acumiatus		EF108155	
Rhinolophus	affinis		EU434934	
Rhinolophus	blasii		EU436669	
Rhinolophus	blythi		DQ865344	
Rhinolophus	borneensis		EF108162	
Rhinolophus	clivosus		EU436674	
Rhinolophus	cornutus		DQ297594	
Rhinolophus	creaghi		EF108164	
Rhinolophus	darlingi		EU436675	
Rhinolophus	eloquens		EU436677	
Rhinolophus	eurvale		EU436671	
Rhinolophus	ferrumequinum		EU436673	
Rhinolophus	formosae		NC_011304	
Rhinolophus	fumigatus		EU436678	
Rhinolophus	hildebrandti		EU436676	
Rhinolophus	hipposideros		EU360631	
Rhinolophus	landeri		FJ457612	
Rhinolophus	lepidus		AF451338	
Rhinolophus	luctus		EF544422	
Rhinolophus	macrotis		EU434957	
Rhinolophus	marshalli		EU434938	
Rhinolophus	mehelyi		EU436672	
Rhinolophus	monocerus		EF555788	
Rhinolophus	pearsonii		EU434940	
Rhinolophus	perditus		AY141039	
Rhinolophus	philippinensis		EF108169	
Rhinolophus	pumilus		NC_005434	
Rhinolophus	pusillus		EF217392	
Rhinolophus	rex		EU075216	
Rhinolophus	sedulus		EF108174	
Rhinolophus	simulator		EU436670	
Rhinolophus	sinicus		EU434941	
Rhinolophus	sp.1KS-2008		EU434937	
Rhinolophus	sp.2KS-2008		EU434942	
Rhinolophus	stheno		EF108175	
Rhinolophus	thomasi		EU434943	
Rhinolophus	trifoliatus		EF108177	
Rhinolophus	xinanzhongguoensis		EU750753	
Rhinophylla	alethina		AF187028	
Rhinophylla	fischerae		AF187032	
Rhinophylla	pumilio		AF187031	
Rhinopoma	hardwickei		AY056462	
Rhinopoma	microphyllum		AM931063	
Rhinopoma	muscatellum		DQ337500	
Rhogeessa	aeneus		EF222359	
Rhogeessa	genowaysi		EF222326	
Rhogeessa	gracilis		EF222412	
Rhogeessa	io		EF222392	
Rhogeessa	mira		EF222336	
Rhogeessa	parvula		EF222355	
Rhogeessa	tumida		EF222367	
Rhogeessa	velilla		EF222341	
Rhynchonycteris	naso		EF584192	
Rousettus	aegyptiacus		EU624124	
Rousettus	aegyptiacus	aegyptiacus	AF044658	
Rousettus	aegyptiacus	princeps	AF044659	
Rousettus	amplexicaudatus		AB046329	
Rousettus	angolensis		AF044643	
Rousettus	lanosus		AF044661	
Rousettus	leschenaultii		FJ549331	
Rousettus	madagascariensis		AF044663	
Rousettus	spinalatus		EF105523	
Saccopterix	bilineata		EF584202	
Saccopterix	canescens		EF584206	
Saccopterix	gymnura		EF584208	
Saccopterix	leptura		EF584216	
Scotomanes	ornatus		DQ435069	
Scotophilus	borbonicus		DQ459067	
Scotophilus	dinganii		EU750999	
Scotophilus	heathii		EU750946	
Scotophilus	kuhlii		EU750931	
Scotophilus	leucogaster		EU750940	
Scotophilus	marovaza		EU750943	
Scotophilus	nigrita		EU750955	
Scotophilus	nux		EU750939	
Scotophilus	robustus		EU750948	
Scotophilus	tandrefana		EU750941	
Scotophilus	viridis		EU750991	
Scotophilus	viridis	nigritellus	EU750976	
Sphaeronycteris	toxophyllum		AY604452	
Stenoderma	rufum		AY604431	
Sturnira	luisi	serotinus	AF435170	
Sturnira	luisi	thomasi	AF435250	
Sturnira	luisi	vulcanensis	AF435251	
Sturnira	aratathomasi		AF435252	
Sturnira	bidens		AF435201	
Sturnira	bogotensis		AF435248	
Sturnira	erythromos		FJ154179	
Sturnira	ludovici		AF435235	
Sturnira	luisi	angeli	AF435158	
Sturnira	luisi	paulsoni	AF435162	
Sturnira	luisi	zygomaticus	AF435159	
Sturnira	magna		AF435180	
Sturnira	mordax		AF435212	
Sturnira	nana		AF435253	
Sturnira	oporaphilum		AF435210	
Sturnira	sp.CAI-2003A		AF435203	
Sturnira	sp.CAI-2003B		AF435204	
Sturnira	lilium		AF187035	
Sturnira	tildae		AF435185	
Syconycteris	sp.		AF044665	
Tadarida	brasiliensis		L19734	
Tadarida	fulminans		EU760911	
Tadarida	teniotis		EU360721	
Taphozous	longimanes		EF584219	
Taphozous	melanopogon		EF584221	
Thyroptera	tricolor		AY621005	
Tomopeas	ravus		L19735	
Tonatia	bidens		FJ155490	
Tonatia	saurophila		FJ155488	
Trachops	cirrhosus		FJ155483	
Triaenops	afer		EU798750	
Triaenops	auritus		DQ005794	
Triaenops	furculus		DQ005845	
Triaenops	persicus		EU798758	
Triaenops	rufus		DQ005771	
Triaenops	sp.PPV-2008		EU798756	
Tylonycteris	pachypus		EF517315	
Uroderma	bilobatum		AY169955	
Uroderma	magnirostrum		FJ154180	
Vampyressa	bidens		AY157055	
Vampyressa	melissa		FJ154185	
Vampyressa	pusilla		DQ312428	
Vampyressa	thyone		DQ312431	
Vampyressa	brocki		DQ312421	
Vampyressa	nymphaea		DQ312418	
Vampyrodes	caraccioli		FJ154184	
Vampyrum	spectrum		FJ155482	
Vespertilio	murinus		AB287359	
Vespertilio	sinensis		AB287362	

## References

[ref-3629532914] Felsenstein J. 1985. Phylogneies and the comparative method. American Naturalist 125: 1-15.

[ref-2838699358] Harvey PH, Pagel MD. 1991. The comparative method in evolutionary biology. New York: Oxford University Press.

[ref-3178070789] May-Collado L, Agnarsson I. 2006. Cytochrome b and bayesian inference of whale phylogeny. Molecular Phylogenetics and Evolution 38: 344-354. 10.1016/j.ympev.2005.09.019 16325433

[ref-1321353247] Agnarsson I, May-Collado LJ. 2008. The phylogeny of Cetartiodactyla: The importance of dense taxon sampling, missing data, and the remarkable promise of cytochrome b to provide reliable species-level phylogenies. Molecular Phylogenetics and Evolution 48: 964-985. 10.1016/j.ympev.2008.05.046 18590827

[ref-1227262516] Agnarsson I, Kuntner M, May-Collado LJ. 2010. Dogs, cats, and kin: A molecular species-level phylogeny of Carnivora. Molecular Phylogenetics and Evolution 54: 726-745. 10.1016/j.ympev.2009.10.033 19900567

[ref-4050571394] Kuntner M, May-Collado L, Agnarsson I. 2011. Phylogeny and conservation priorities of afrotherian mammals (Afrotheria, Mammalia). Zoologica Scripta 40: 1-15.

[ref-390496433] Bininda-Emonds ORP. 2005. Supertree construction in the genomic age. In E. A. Zimmer & E. H. Roalson (Eds) Molecular evolution: Producing the biochemical data, part b, methods in enzymology pp. 745-757. Elsevier. 10.1016/S0076-6879(05)95038-6 15865993

[ref-4258870179] Lapointe FJ, Kirsch JA, Hutcheon JM. 1999. Total evidence, concensus, and bat phylogeny: a distance-based approach. Molecular Phylogenetics and Evolution 11: 55-66. 10.1006/mpev.1998.0561 10082610

[ref-3848735776] Simmons NB, Geisler JH. 2002. Sensitivity analysis of different methods of coding taxonomic polymorphism: an example from higher-level bat phylogeny. Cladistic 18: 571-584.

[ref-3687832270] Giannini NP, Simmons NB. 2003. A phylogeny of megachiropteran bats (Mammalia: Chiroptera: Pteropodidae) based on direct optimization analysis of one nuclear and four mitochondrial genes. Cladistics 19: 496-511. 10.1111/j.1096-0031.2003.tb00385.x 34905855

[ref-1849758586] Piaggio AJ, Perkins SL. 2005. Molecular phylogeny of North American long-eared bats (Vespertillionidae: Corynorhinus); inter and intraspecific relationships inferred from mitochondrial and nuclear DNA sequences. Molecular Phylogenetics and Evolution 37: 762-775. 10.1016/j.ympev.2005.03.029 15869885

[ref-3002310841] Hoffmann FG, Hoofer SR, Baker RJ. 2008. Molecular dating of the diversification of Phyllostominae bats based on nuclear and mitochondrial DNA sequences. Molecular Phylogenetics and Evolution 49: 653-658. 10.1016/j.ympev.2008.08.002 18727956

[ref-1881891542] Jones KE, Purvis A, MacLarnon A, Bininda-Emonds ORP, Simmons NB. (2002). A phylogenetic supertree of the bats (Mammalia: Chiroptera). Biological Reviews, 77, 223-259. 10.1017/s1464793101005899 12056748

[ref-3868830572] Tobe SS, Kitchener AC, Linacre AMT. 2010. Reconstructing mammalian phylogenies: a detailed comparison of the cytochrome b and cytochrome oxidase subunit I mitochondrial genes. PLoS ONE 5(11): e14156. 10.1371/journal.pone.0014156 PMC2994770 21152400

[ref-65794393] Nishihara H, Hasegawa M, Okada N. 2006. Pegasoferae, an unexpected mammalian clade revealed by tracking ancient retroposon insertions. Proceedings of the National Academy of Sciences of the United States of America 103: 9929-9934. 10.1073/pnas.0603797103 PMC1479866 16785431

[ref-1405340400] Maddison WP. 1993. Missing data versus missing characters in phylogenetic analysis. Systematic Biology 42: 576-581.

[ref-537799374] Maddison WP, Maddison DR. 2010. Mesquite: A modular system for evolutionary analysis. Ver. 2.74 build 550. Available at: http://mesquiteproject.org.

[ref-722950374] Posada D. jModelTest: phylogenetic model averaging. Mol Biol Evol. 2008 Jul;25(7):1253-6. Epub 2008 Apr 8. 18397919 10.1093/molbev/msn083

[ref-1393364660] Posada D, Buckley TR. 2004. Model selection and model averaging in phylogenetics: Advantages of the aic and bayesian approaches over likelihood ratio tests. Systematic Biology 53: 793-808. 10.1080/10635150490522304 15545256

[ref-82833293] Rodríguez F, Oliver JF, Marín A, Medina JR. 1990. The general stochastic model of nucleotide substitution. Journal of Theoretical Biology 142: 485-501. 10.1016/s0022-5193(05)80104-3 2338834

[ref-3109912983] Yang Z. 1994. Maximum likelihood phylogenetic estimation from DNA sequences with variable rates over sites: approximate methods. Journal of Molecular Evolution 39: 306-314. 10.1007/BF00160154 7932792

[ref-351852703] Huelsenbeck J P, Ronquist F. 2001. Mrbayes: Bayesian inference of phylogenetic trees. Bioinformatics 17: 754-755. 10.1093/bioinformatics/17.8.754 11524383

[ref-938599339] Drummond AJ, Ho SYW, Phillips MJ, Rambaut A. 2006. Relaxed phylogenetics and dating with confidence. PLoS Biology 4: e88. 10.1371/journal.pbio.0040088 PMC1395354 16683862

[ref-639092272] Drummond AJ, Rambaut A. 2007. BEAST: Bayesian evolutionary analysis by sampling trees. BMC Evolutionary Biology 7: 214. 10.1186/1471-2148-7-214 PMC2247476 17996036

[ref-2894200076] Revilliod P. 1920. Contribution a L’étude des Chiroptères des terrains tertiaires. Mémoires de la Société Paléontologique Suisse part II 44.

[ref-2394571077] Stoffberg S, Jacobs DS, Mackie IJ, Matthee CA. 2010. Molecular phylogenetics and historical biogeography of Rhinolophus bats. Molecular Phylogenetics and Evolution 54: 1-9. 10.1016/j.ympev.2009.09.021 19766726

[ref-186333871] Cao Y, Fujiwara M, Nikaido M, Okada N, Hasegawa M. 2000. Interordinal relationships and timescale of eutherian evolution as inferred from mitochondrial genome data. Gene 259: 149-158. 10.1016/s0378-1119(00)00427-3 11163972

[ref-1120880631] Eiting TP, Gunnell GF. 2009. Global completeness of the bat fossil record. Journal of Mammalian Evolution 16:151–173

[ref-3006004211] Springer MS, DeBry RW, Douady C, Amrine HM, Madsen O, de Jong WW, Stanhope MJ. 2001. Mitochondrial versus nuclear gene sequences in deep-level mammalian phylogeny reconstruction. Molecular Biology and Evolution 18: 132-143. 10.1093/oxfordjournals.molbev.a003787 11158372

[ref-240486202] Hoofer SR, Reeder SA, Hansen EW, Van den Bussche RA. 2003. Molecular phylogenetics and taxonomic review of noctilionoid and vespertilionoid bats (Chiroptera: Yangochiroptera). Journal of Mammalogy 84: 809-821.

[ref-309990588] Van den Bussche RA, Hoofer SR, Simmons NB. 2002. Phylogenetic relationships of mormoopid bats using mitochondrial gene sequence and morphology. Journal of Mammalogy 83: 40-48.

[ref-3144902686] Teeling EC, Madsen O, Stanhope MJ, de Jong WW, Van Den Bussche R, Springer MS. 2002. Microbat paraphyly and the convergent evolution of a key innovation in Old World rhinolophoid microbats, Proceedings of the National Academy of Sciences USA 99: 1432-1436. 10.1073/pnas.022477199 PMC122208 11805285

[ref-12631350] Teeling EC, Madsen O, Murphy WJ, Springer MS, O’Brien JO. 2003. Nuclear gene sequences confirm an ancient link between New Zealand’s short tailed bat and South American noctilionoid bats. Molecular Phylogenetics and Evolution 28: 308-319. 10.1016/s1055-7903(03)00117-9 12878467

[ref-25426364] Teeling EC, Springer MS, Madsen O, Bates P, O’Brien SJ, Murphy WJ. 2005. A molecular phylogeny of bats illuminates biogeography and fossil record. Science 307: 580-584. 10.1126/science.1105113 15681385

[ref-4210006386] Lim BK. 2007. Divergence times and origin of neotropical sheath-tailed bats (Tribe Diclidurini) in Southe America. Molecular Phylogenetics and Evolution 45: 777-791. 10.1016/j.ympev.2007.09.003 17937995

[ref-2216998409] Kennedy M, Paterson AM, Morals JC, Parsons S, Winnington AP, Spencer HG. 1999. The long and short of it: branch lengths and the problem of placing the New Zealand short-tailed bat, Mystacina. Molecular Phylogenetics and Evolution 13: 405-416. 10.1006/mpev.1999.0660 10603267

[ref-520085304] Arnason U, Adegoke JA, Gullberg A, Harley EH, Janke A, Kullberg M. 2008. Mitogenomic relationships of placental mammals and molecular estimates of their divergences. Gene 421: 37-51. 10.1016/j.gene.2008.05.024 18590805

[ref-1187845697] Jones KE, Bininda-Emonds ORP, Gittleman JL. 2005. Bats, clocks, and rocks: diversification patterns in Chiroptera. Evolution 59: 2243-2255 16405167

